# Conducting Polymer‐Ionic Liquid Electrode Arrays for High‐Density Surface Electromyography

**DOI:** 10.1002/adhm.202100374

**Published:** 2021-05-14

**Authors:** Santiago Velasco‐Bosom, Nuzli Karam, Alejandro Carnicer‐Lombarte, Johannes Gurke, Nerea Casado, Liliana C. Tomé, David Mecerreyes, George G. Malliaras

**Affiliations:** ^1^ Electrical Engineering Division University of Cambridge Cambridge CB3 0FA UK; ^2^ POLYMAT University of the Basque Country UPV/EHU Avda. Tolosa 72, Donostia‐San Sebastián Gipuzkoa 20018 Spain; ^3^ Ikerbasque Basque Foundation for Science Bilbao E‐48011 Spain; ^4^ Present address: LAQV/REQUIMTE, Chemistry Department NOVA School of Science and Technology Caparica 2829‐516 Portugal

**Keywords:** cutaneous electrophysiology, electromyography, organic bioelectronics, poly(3,4‐ethylenedioxythiophene) polystyrene sulfonate

## Abstract

Surface electromyography (EMG) is used as a medical diagnostic and to control prosthetic limbs. Electrode arrays that provide large‐area, high density recordings have the potential to yield significant improvements in both fronts, but the need remains largely unfulfilled. Here, digital fabrication techniques are used to make scalable electrode arrays that capture EMG signals with mm spatial resolution. Using electrodes made of poly(3,4‐ethylenedioxythiophene) polystyrene sulfonate (PEDOT:PSS) composites with the biocompatible ionic liquid (IL) cholinium lactate, the arrays enable high quality spatiotemporal recordings from the forearm of volunteers. These recordings allow to identify the motions of the index, little, and middle fingers, and to directly visualize the propagation of polarization/depolarization waves in the underlying muscles. This work paves the way for scalable fabrication of cutaneous electrophysiology arrays for personalized medicine and highly articulate prostheses.

## Graphical abstract

The development of easy to fabricate and customize cutaneous electrophysiology electrode arrays for high spatial resolution recordings is reported. Their mechanical flexibility and non‐drying electrodes allow the acquisition of high‐quality signals. These devices have the potential to enable personalized medicine and highly articulated myoelectric prostheses.

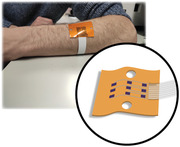

## 1 Introduction

Current developments in electromyography (EMG) focus on the development of better tools for the diagnosis of (neuro‐)muscular pathologies and control of myoelectric prostheses.^[^ ^1^, ^2^, ^3^, ^4^ ^]^ Whereas needle EMG provides very high quality and locally specific signals, the insertion of these electrodes in the muscle and the pain associated with the process, make this technology unsuitable for long‐term use.^[^ ^5^ ^]^ Alternatively, less invasive approaches rely on microneedle devices that penetrate the epidermal barrier to improve the electrical contact between device and skin.^[^ ^6^ ^]^ However, the fabrication processes of these devices impede large area scalability, and the fact that they still penetrate the epidermis makes them uncomfortable to wear and carries the risk of infections.^[^ ^7^ ^]^ On the contrary, surface electrodes have proven to be a more convenient approach for prosthetic control, as they can be used in a non‐invasive configuration and still provide significant information on the neuromuscular system.^[^ ^2^, ^4^ ^]^ The development of surface electrodes is benefiting from advances in the fabrication of mechanically flexible, large area and wearable devices that intimately adjust to the contours of the body, targeting reliable long term recordings of vital signals.^[^ ^8^, ^9^, ^10^, ^11^, ^12^, ^13^, ^14^ ^]^

Electrode arrays that record high density EMG signals are much needed tools for diagnostic purposes and to accurately estimate movement intention and drive prostheses.^[^ ^14^ ^]^ Previous research toward this goal includes the development of a 7 × 13 electrode array of Ag/AgCl electrodes with a diameter of 1.95 mm placed on a 4 mm center‐to‐center distance.^[^ ^15^ ^]^ The electrodes were patterned on a flexible Kapton substrate that conformed well to the skin. However, the skin attachment process was labor‐intensive and required the use of a pre‐patterned adhesive tape that was first attached to the skin and a conductive cream for improved electrical contact. A 5 × 5 stretchable array of Au electrodes with a diameter of 4 mm and center‐to‐center distance of 10 mm was fabricated using serpentine connections and used to control home electronics using EMG signals.^[^ ^16^ ^]^ Another approach explored temporary tattoo electrodes made of conducting polymer to achieve stable, low impedance contact to skin.^[^ ^17^ ^]^ Arrays featuring electrodes with a diameter as small as 5 mm were shown to acquire EMG signals from the cheek of volunteers and identify different facial expressions. In another report, a 4 × 7 array of conducting polymer electrodes with 16 mm^2^ areas was inkjet printed on Kapton and shown to capture EMG activity from the biceps of a volunteer.^[^ ^18^ ^]^ More recently, stretchable arrays of tattoo‐like electrodes that conform over large areas of the body were demonstrated.^[^ ^13^, ^19^ ^]^ The electrodes were made of Au with typical dimensions of 1 × 1 cm^2^ and were connected to electronics with serpentine tracks to allow stretchability. The acquired EMG signals were used to demonstrate control of a prosthetic arm.

In addition to obtaining signals from large areas, it is important to obtain signals at high spatial resolution, to enable non‐invasive studies of (neuro‐)muscular disorders and more precise control of myoelectric prostheses. However, when miniaturizing electrodes, their high impedance can become a problem.^[^ ^20^ ^]^ Conductive polymers such as the commercially available poly(3,4‐ethylenedioxythiophene) polystyrene sulfonate (PEDOT:PSS) have emerged as excellent candidates for microfabricated electrodes, as their volumetric capacitance helps lower the impedance of metal electrodes.^[^ ^21^ ^]^ PEDOT:PSS electrodes have also been used in cutaneous electrophysiology.^[^ ^22^, ^23^, ^24^ ^]^ As a further development, it was shown that PEDOT:PSS electrodes coated with ionic liquid gels form good mechanical and electrical contact to skin and enable long term, high quality cutaneous recordings.^[^ ^25^, ^26^ ^]^

Recently, the incorporation of ionic liquids (ILs) into PEDOT:PSS was explored as a means to increase electronic conductivity and tune mechanical properties.^[^ ^27^ ^]^ The use of such PEDOT:PSS:IL composites as electrodes for cutaneous electrophysiology would thus be an interesting strategy to achieve good contact to skin and simplify device fabrication. In this paper, we show a new, scalable fabrication process for making mm‐sized cutaneous arrays of PEDOT:PSS:IL electrodes. The arrays record high quality spatiotemporal EMG maps, paving the way for improved and customizable medical diagnostics and myoelectric prostheses.

## 2 Results and Discussion

**Figure 1:**
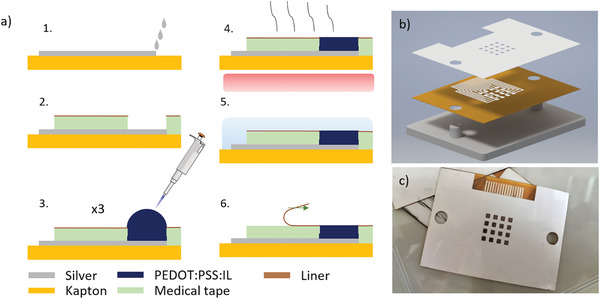
a) Fabrication scheme. 1) Inkjet printing of silver tracks, 2) attachment of the patterned medical tape, 3) deposition of PEDOT:PSS:IL, 4) baking, 5) soaking in saline, and 6) removal of the top liner. b) Exploded view of the device assembly process showing the aligning holder. c) Photo of a device before peel‐off of the top liner and attachment of the cable.

The electrode array fabrication process is shown in **Figure** **1**a, with details given in the Experimental Section. Metal tracks are deposited on a 50 µm thick polyimide (Kapton) substrate by inkjet printing a silver nanoparticle ink. The substrate is then mounted on an aligning holder (Figure 1b). A 120 µm thick double‐sided medical‐grade tape is patterned by laser to define a 4 × 4 array of 1.5 by 1.5 mm^2^ through holes with a center‐to‐center distance of 3 mm. The tape is chosen for its skin compatibility and long‐term adhesion properties. The bottom liner is peeled‐off, and the tape is adhered on the Kapton substrate with the aid of the aligning holder. This brings the through holes on the tape in registry with the metal tracks on the Kapton and defines wells into which the PEDOT:PSS:IL mixture is drop cast. We use the cholinium lactate ionic liquid (Figure S1, Supporting Information) due to its high biocompatibility.^[^ ^28^ ^]^ The amount of solution cast was calibrated to not overflow the wells. The device is subsequently baked to remove solvents and immersed in deionized (DI) water. The final array is shown in Figure 1c. Connection to recording electronics is achieved through a flat cable, attached on the array by anisotropic conducting film (ACF). The top liner is peeled‐off before application to skin, exposing an adhesive surface between the electrodes. The medical tape, therefore, serves multiple roles: insulating the silver tracks, defining the PEDOT:PSS:IL electrode geometry, and providing adhesion to skin. The overall thickness of the array is 170 µm, enabling excellent conformability to the contours of the human body.

**Figure 2:**
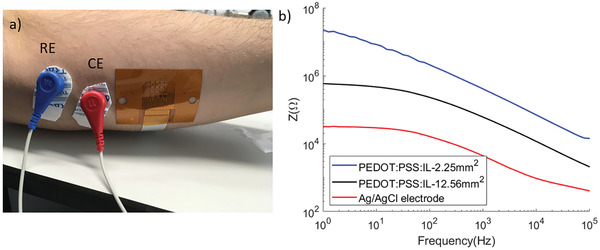
a) Location of the electrode array, the reference electrode, and counter electrode. b) Impedance spectra of PEDOT:PSS:IL electrodes with different areas (blue for 2.25 mm^2^, black for 12.56 mm^2^) compared to a commercial Ag/AgCl electrode (314 mm^2^).

The impedance of the PEDOT:PSS:IL electrodes attached on the arm of volunteers was measured with Electrochemical Impedance Spectroscopy (EIS). All experiments were performed with the approval of the local Ethics Committee and after obtaining informed consent from volunteers. A 3‐electrode configuration was used (**Figure** **2**a), with the electrode arrays located on the inner part of the left forearm, and with commercial Ag/AgCl electrodes used as reference (RE) and counter electrodes (CE). At 60 Hz, which is in the middle of the EMG signal range,^[^ ^2^ ^]^ the 1.5 mm by 1.5 mm PEDOT:PSS:IL electrodes with an area of 2.25 mm^2^ have an impedance of 2.7 MΩ (average of *N* = 12 electrodes), while PEDOT:PSS:IL electrodes with an area 12.56 mm^2^ have an impedance of 0.3 MΩ (average of *N* = 4 electrodes). For comparison, the EIS spectrum of a commercial Ag/AgCl electrode, placed on the same location as the electrode array, is also shown in Figure 2. The Ag/AgCl electrode represents the clinical standard, and when measured under the same configuration, it has an impedance of 17 kΩ at 60 Hz. However, the lower impedance of the Ag/AgCl electrode is partly due to its larger area (314 mm^2^). Indeed, Ag/AgCl electrodes with an area of 12 mm^2^, assisted by conducting creme and placed on skin rubbed with alcohol, show impedances in the range of ≈120 kΩ at 62.5 Hz.^[^ ^15^ ^]^ PEDOT:PSS is known to be a volumetric capacitor, with an effective capacitance per unit area that largely exceeds that of polarizable metal electrodes.^[^ ^29^ ^]^ Normalized per area, the PEDOT:PSS:IL electrodes show an impedance of 48 kΩ∙cm^2^ at 60 Hz (average of *N* = 16 electrodes). This is in the same range as values reported for cutaneous PEDOT:PSS electrodes (≈15 kΩ∙cm^2^ at 60 Hz),^[^ ^18^ ^]^ PEDOT:PSS electrodes coated with an ionic liquid gel (≈50 kΩ∙cm^2^ at 60 Hz),^[^ ^25^ ^]^ and electrically conductive Ecoflex composites (≈50 kΩ∙cm^2^ at 60 Hz).^[^ ^11^ ^]^ These values should be taken as order‐of‐magnitude estimates, as impedance varies with skin type, preparation, and level of hydration.

**Figure 3:**
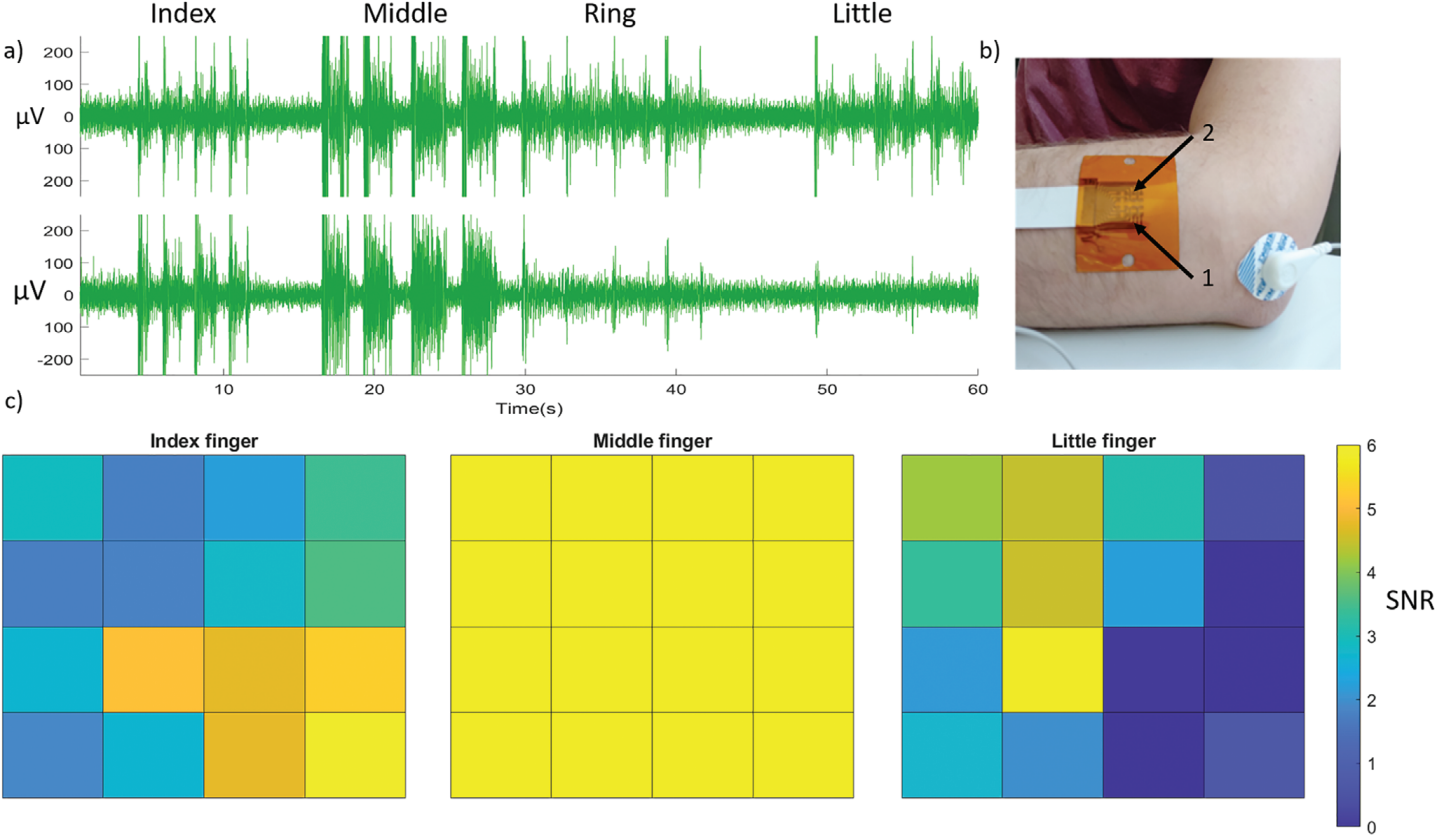
a) EMG recordings from two electrodes during the motion of different fingers. b) Image showing the location of the electrode array and the reference electrode. The arrows indicate the position of the two electrodes in (a), over the extensor carpi ulnaris (arrow #1) and the extensor digitorum communis (arrow #2) muscles, respectively. c) Heat maps showing the normalized signals recorded by the array during the motion of different fingers. Arrow #1 in (b) points to the third row, first column electrode of the heatmap, while arrow #2 points to the third row, fourth column electrode of the heatmap.

To demonstrate the ability of the electrodes to record EMG signals, arrays were placed on the posterior forearm of a volunteer, over the *extensor digitorum communis* and the *extensor carpi ulnaris* muscles (**Figure** **3**a). Starting from a resting position with the palm facing downward, the volunteer was asked to raise four times each finger (index, middle, ring, and little fingers) individually before returning to a relaxed position. The biological origin of the recorded signals was validated by the simultaneity of the action performed with the corresponding recording. An example of complete recordings is shown in Figure S2, Supporting Information. In Figure 3a, recordings from two electrodes are shown. The first electrode is located over the *extensor carpi ulnaris* muscle, which controls the movement of the little finger, while the second electrode is placed over the *extensor digitorum communis* muscle, which controls the movement of the index finger. The location of these two electrodes on the arm is indicated by arrows in Figure 3b. Both electrodes show the typical EMG bursts when fingers are moved. Importantly, clear selectivity is demonstrated, with electrode 1 showing proportionately higher signal when the little finger is moved, and electrode 2 showing proportionately higher signal when the index finger is moved. Movement of the middle and ring fingers causes the same signal to be recorded on both traces, as the *extensor digitorum communis* muscle that controls these fingers lies under both electrodes.

The spatial selectivity is also seen on the heat maps that plot signal‐to‐noise ratio (SNR) measured during the motion of different fingers (Figure 3c). For this analysis, the initial spikes observed in the first 0.2 s of the event are omitted, as they might be due to an artifact arising from device movement.^[^ ^2^ ^]^ The SNR is calculated as the ratio of the root mean squared (RMS) of the measured voltage over a period of 0.5 s preceding the initial spikes, divided by the RMS of the signal measured when the finger is at rest. Movement of the index finger predominantly activates electrodes located on the bottom‐right of these maps, whereas movement of the little finger activates electrodes on the top‐left. On the other hand, when the middle finger is moved, an intense signal (SNR = 6, or 15.6 dB) is recorded by all electrodes, as the *extensor digitorum communis* muscle underlies the entire array. A similar map was observed when the ring finger was moved, as this finger is also controlled by the *extensor digitorum communis* muscle. The SNR values shown in Figure 3c are large enough to be of use in state‐of‐the‐art myoelectric prostheses.^[^ ^30^, ^31^ ^]^ For comparison, cm‐scale Ag/AgCl electrodes and electrodes made of polyurethane nanofibers and Ag nanowires record EMG signals with an SNR of ≈28 dB.^[^ ^12^ ^]^

**Figure 4:**
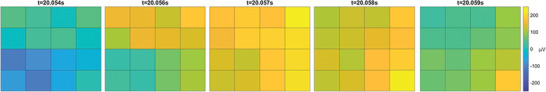
Temporal evolution of the recorded voltage during movement of the middle finger. The orientation of these maps is the same as in Figure 3c.

The arrays provide spatiotemporal maps of the progression of polarization and depolarization in fiber bundles of the underlying muscle in a non‐invasive fashion. This is shown in **Figure** **4**, where five snapshots of the recorded voltage during the movement of the middle finger are shown. These maps show a polarization wave sweeping in from the top right corner, followed by a depolarization wave that appears from the top left corner. A longer sequence is available for viewing as a movie in Supporting Information (Video SV1). These maps and the ones of Figure 3c, clearly demonstrate that electrodes placed at a center‐to‐center distance of 3 mm record spatially different information. Such high‐resolution spatial maps are useful in the clinic to help determine muscle conduction velocity, estimate the size and number of motor units, and investigate (neuro‐)muscular pathologies.^[^ ^32^ ^]^ They can also be used to train classifiers that learn to discriminate between different intended motions for highly articulate myoelectric prostheses.^[^ ^30^ ^]^

The results shown here indicate that PEDOT:PSS:IL forms good enough mechanical and electrical contact to skin to allow high quality EMG recordings to be made from mm‐size electrodes. Despite the fact that their impedance is in the MΩ range, they are able to record with an SNR as high as 6, enabling the simple fabrication of surface arrays with mm spatial resolution. The 4 × 4 array demonstrated here obtains high‐resolution spatiotemporal maps that are of interest for diagnostic applications and for driving highly articulate myoelectric prostheses. The incorporation of a biocompatible IL inside the PEDOT:PSS structure simplifies fabrication compared to previous works where an IL gel was deposited over a PEDOT:PSS electrode.^[^ ^25^ ^]^ The resulting electrode is simple to deposit using solution techniques and requires mild post‐deposition treatment, making it compatible with flexible substrates such as Kapton. Moreover, no conducting gels or cremes are needed to enable skin contact.

The fabrication process described here defines the array geometry using the digital techniques of inkjet printing and laser cutting. The deposition of PEDOT:PSS:IL, which was carried out here using drop casting, can be achieved using large area techniques such as spraying or bar coating. The removal of the top liner can then serve as a lift‐off step that patterns the conducting polymer. A similar process involving mechanical peel‐off of a sacrificial layer has been demonstrated in micron scale devices.^[^ ^33^ ^]^ The array geometry can thus be easily modified, without the need to change any hard (physical) masks. This paves the way for customization, a key objective of personalized medicine, and an enabler for more performant myoelectric prostheses. Moreover, fabrication can be easily scaled to large areas: Even with the most basic laboratory equipment, the fabrication of arrays with sizes as large as A4 is feasible. Such large arrays placed over muscles, the heart, or the brain, have the potential to revolutionize non‐invasive diagnostics and enable a new era in brain‐computer interfaces.

Finally, it should be mentioned that the arrays reported in this work are flexible but not stretchable. There is currently a great deal of recent interest in making stretchable electrodes that integrate with the skin, often in a tattoo‐like fashion. Compared to these, the arrays presented here might appear less technologically sophisticated. However, in most applications of interest to clinical EMG and control of myoelectric prostheses, electrodes are placed on areas of the body where the skin does not deform enough to necessitate the use of stretchable electrodes. This is especially true when the electrodes (and hence the resulting array) are small. Moreover, tattoo‐like electrodes are not as straightforward to apply to the skin and to connect to, while the arrays reported here are easy to handle and can be connected to electronics using commercial ACF technology. The solution we present here is hence practical and addresses the unmet healthcare need for high resolution EMG.

## 3 Conclusions

We show here a new fabrication method for obtaining adhesive electrode arrays for cutaneous electrophysiology. Using the digital techniques of inkjet printing and laser cutting, we demonstrate a 4 × 4 array of 1.5 × 1.5 mm^2^ electrodes with a center‐to‐center distance of 3 mm. PEDOT:PSS composites with the biocompatible ionic liquid cholinium lactate are used as electrodes, showing an impedance of 2.7 MΩ at 60 Hz. The array enables high quality spatiotemporal recordings of EMG that allow to identify the motions of the index, little and middle fingers, with an SNR of 6 for the latter. Moreover, they allow to directly visualize the propagation of polarization/depolarization waves in the underlying muscles. This work paves the way for scalable fabrication of cutaneous electrophysiology arrays, with obvious applications in the monitoring of muscles, the heart, the brain, and other electrically active organs. It supports the development of customizable arrays for personalized medicine and enables for highly articulate myoelectric prostheses.

## 4 Experimental Section

### Fabrication of Electrodes

Silver nanoparticle ink (Sicrys 130EG‐1) was degassed, sonicated (5 min) and filtered (PTFE 0.45 µm) before insertion into a Dimatix cartridge (11610, 10 pl nozzle to print Ag tracks on a polyimide substrate (Kapton 54 × 40 mm, 50 µm thick) using a PiXDRO LP50. The Kapton substrate was laser cut (VLS 2.30, Universal Laser Systems) to shape with markers for subsequent alignment, then sonicated in DI water and wiped gently to remove debris from the cutting process. Two layers of silver were printed (800 dpi and 2000 dpi) to obtain improved area coverage and conductivity. The ink was left to dry (40 °C) before applying thermal sintering (120 °C, 60 min) in a conventional oven, according to the manufacturer's specifications.

For the assembly and insulation of the device, double‐side adhesive medical tape (120 µm thick) was laser cut in registry with the tracks on the Kapton substrate. The bottom liner was removed and the medical tape was adhered to the substrate, leaving the electrodes and external connections exposed. The assembly process was carried out with the aid of an aligning holder. For the PEDOT:PSS:IL films, PEDOT:PSS (Heraeus Clevios 1 wt%) was mixed (40 wt%) with cholinium lactate (IOLITEC) and (3‐glycidyloxypropyl)trimethoxysilane (GOPS, 1 wt%) and stirred thoroughly using a vortex mixer. It was filtered (PTFE 0.45 µm) and drop cast (1.8 µL) on each well defined by the double adhesive tape and left to dry overnight. This process was repeated three times to obtain a thick layer that did not overflow the well. The films were hard‐baked (130 °C, 60 min) and immersed overnight in deionized water to remove the unwanted excess low molecular weight compounds. ACF bonding was used to connect the device to a commercial external zero insertion force (ZIF) connector that matched the 500 µm width of the tracks.

### In Vivo Recordings

All experiments were performed with the approval of the Ethics Committee of the Department of Engineering at the University of Cambridge (6/9/2018, IONBIKE) and after obtaining informed consent from volunteers. The skin was prepared by gentle wiping with a tissue wet in ethanol. The top liner of the device was removed before attaching it to the skin. A period of 5 min took place between the adhesion of the electrodes and any recordings, to provide enough time for stabilization of the electrode/skin interface. For the impedance measurements, a PGSTAT128N Metrohm AUTOLAB potentiostat was used, with two commercial Ag/AgCl electrodes (MLA1010B, ADInstruments) as reference and counter electrodes. For the EMG recordings, a commercial Ag/AgCl electrode was used as reference. All 16 electrodes were recorded simultaneously using a RHS stimulation and recording system from Intan technologies. The sampling rate was 30 kHz. The acquired signals were filtered using a 50 Hz band‐stop filter, and a band pass filter with cut off frequencies of 10 Hz and 100 Hz.

## Conflict of Interest

The authors declare no conflict of interest.

## Supporting information

Supporting Information

## Data Availability

Data available on request from the authors.
