# Supplementary material for: Conducting Polymer‐Ionic Liquid Electrode Arrays for High‐Density Surface Electromyography
Source: Adv Healthc Mater. 2021 May 14;10(17):2100374. doi: 10.1002/adhm.202100374 (PMC11469138; doi:10.1002/adhm.202100374)
Supplement: Supplementary file 1 — Supporting Information [file ADHM-10-2100374-s001.pdf]

© 2021 Wiley-VCH GmbH

ADVANCED HEALTHCARE MATERIALS

Supporting Information

for *Adv. Healthcare Mater.*, DOI: 10.1002/adhm.202100374

Conducting Polymer-Ionic Liquid Electrode Arrays for High-Density Surface Electromyography

*Santiago Velasco-Bosom, Nuzli Karam, Alejandro Carnicer-Lombarte, Johannes Gurke, Nerea Casado, Liliana C. Tomé, David Mecerreyes, and George G. Malliaras**

Supporting Information

Conducting Polymer-Ionic Liquid Electrode Arrays for High-Density Surface Electromyography

Santiago Velasco-Bosom,[1] *Nuzli Karam*[1], *Alejandro Carnicer-Lombarte,*[1] *Johannes Gurke,*[1] *Nerea Casado,*[2] *Liliana C. Tomé,*[2,†] *David Mecerreyes,*[2,3] *George G. Malliaras*[1,*]

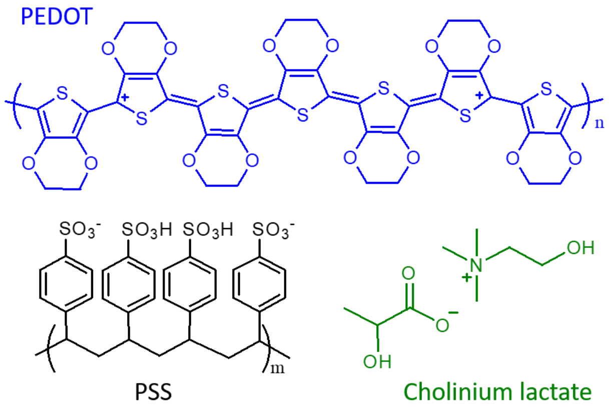

Figure S1: Chemical structures of PEDOT:PSS and IL cholinium lactate.

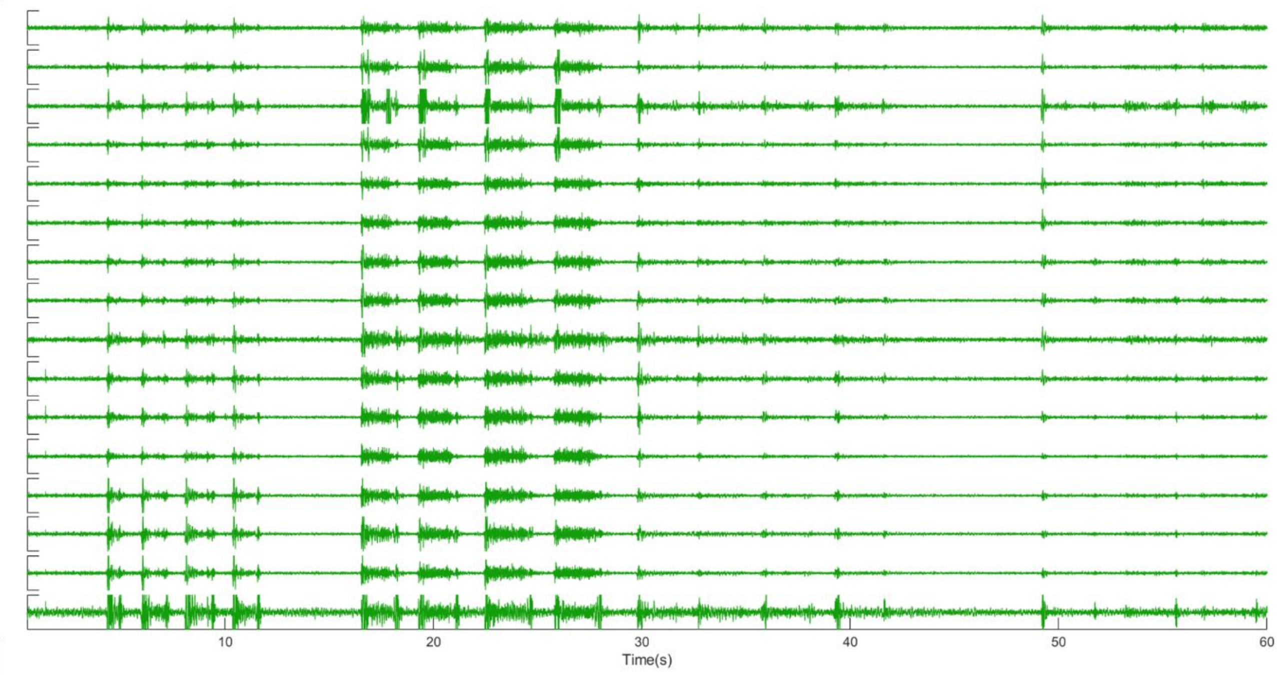

Figure S2: Example of recordings during finger contractions from all electrodes. Y axis ranges from -250 to 250 μV. The first four recordings correspond to electrodes of row 1, column 1-4 and so on.

Video SV1: Temporal evolution of the recorded voltage for all the electrodes during movement of the middle finger. The sampling rate of raw signal was 30 kHz. For visual purposes the heatmap shows one every ten samples.
